# Retraction: Social success of perfumes

**DOI:** 10.1371/journal.pone.0222524

**Published:** 2019-09-10

**Authors:** The PLOS ONE Editors

After this article [1] was published, questions were raised about the dataset used in the study. In following up on these questions it came to light that the dataset was obtained from a third-party commercial entity whose identity cannot be shared due to a nondisclosure agreement, and that the authors cannot share the raw data or provide clarifications about how the data were collected or processed. The authors posted anonymized summary data on Figshare, as noted in the article’s Data Availability Statement. However, the reported Methods are not sufficient to enable other researchers to reproduce the study and the data provided do not meet *PLOS ONE*’s requirements as outlined in our Data Availability policy. The authors noted that they cannot reproduce the analyses using another public dataset as no comparable dataset is currently available.

In light of these issues, the *PLOS ONE* Editors retract this article due to concerns about the reproducibility of the study and noncompliance with the journal’s Data Availability policy. We regret that these issues were not identified prior to the article’s publication.

VV and TSE agreed with retraction.

## References

[1] Vasiliauskaite V, Evans TS (2019) Social success of perfumes. PLoS ONE 14(7): e0218664 10.1371/journal.pone.0218664 31269036 PMC6609013

